# Dr. Wilson on the Cure of Neuralgia

**Published:** 1829-01-01

**Authors:** 


### XXXV.

### NEURALGIA.

Dr. Wilson, the talented author of a work on West India Fever, reviewed in a former Number of this Journal, has transmitted to us some observations on the dreadful case of Neuralgia lately published for consultation, in No. XVIII. of this Journal, page 435. We shall take the liberty of quoting a passage from Dr. Wilson's letter, as it contains some practical facts of interest and utility.

"In three cases of neuralgic disease lately under my care, a cure was effected by the combination of calomel, opium, and oil of turpentine. In my Memoirs of West India Fever, I there stated my belief that the oil of turpentine not only accelerated the action of mercury, but, also, in certain cases, increased its curative effects. In the neuralgic cases to which I allude, I gave the turpentine chiefly with the view of speedily inducing the specific action of the mercury. I am now, however, inclined to believe that the turpentine operated beneficially in itself. The following is the mode of administration which I have employed in the three neuralgic cases above-mentioned.

"A pill, containing from two to four grains of calomel, and one or two grains of opium, was given each night at bedtime, and, next morning, one or two drachms of oil of turpentine, mixed with a little honey. In each of the three cases, a complete and permanent cure has been effected by this plan, and in a moderate space of time."

---

